# Femoral head fracture with large crushed defect in weight-bearing area treated with autologous osteochondral transplantation (repositionplasty): A case report

**DOI:** 10.1097/MD.0000000000032569

**Published:** 2022-12-30

**Authors:** Hyun-Chul Shon, Eic-Ju Lim, Jae-Young Yang, Seung-Jun Jeon

**Affiliations:** a Department of Orthopaedic Surgery, Chungbuk National University Hospital, Cheongju, Korea.

**Keywords:** femoral head fracture, hip dislocation, large defect, osteochondral autologous transplantation, repositionplasty

## Abstract

Femoral head fracture is a rare type of fracture commonly associated with hip dislocation. Femoral head fracture treatments include conservative treatment, fragment excision, open reduction, internal fixation, and arthroplasty. In cases of young patients, open reduction and internal fixation are preferred over arthroplasty. Pipkin type 2 fractures are treated with open reduction and internal fixation in most cases because the fracture line extends upward of the fovea and includes the femoral head-acetabular weight-bearing joint surface. However, if the fracture is severely comminuted or crushed, good results are difficult to obtain with open reduction and internal fixation.

**Patient concerns:**

A 23-years-old male patient presented to the emergency room with right hip pain after 4-m fall

**Diagnosis:**

In initial image work up, he was diagnosed with right posterior hip dislocation and a Pipkin type 2 femoral head fracture with an ipsilateral superior ramus fracture. After manual reduction, simple radiography and computed tomography showed reduction of the hip joint and a large osteochondral defect of approximately 6 cm2 in the weight-bearing area on the superomedial side of the femoral head

**Interventions:**

This was treated with a novel surgery named autologous osteochondral transplantation (repositionplasty), devised by the authors

**Outcomes:**

The congruent reduction was confirmed by radiography and computed tomography immediately after the surgery. At 5 years postoperatively, the Harris Hip Score was 100 points and no discomfort, femoral head collapse, osteonecrosis, or traumatic arthritis were observed on follow-up radiographs

**Lessons:**

We think this method “repositionplasty” will be a good treatment method for young patients with a large defect in the weight-bearing part of the femoral head who cannot undergo open reduction and internal fixation.

## 1. Introduction

Femoral head fracture is a rare type of fracture commonly associated with hip dislocation and occurring in approximately 6% to 15% of posterior hip dislocations.^[1–3]^

Femoral head fracture treatments include conservative treatment, fragment excision, open reduction, internal fixation, and arthroplasty. In cases of femoral head fractures in young and active patients, open reduction and internal fixation are preferred over arthroplasty.^[4]^ Pipkin type 2 fractures are treated with open reduction and internal fixation in most cases because the fracture line extends upward of the fovea and includes the femoral head-acetabular weight-bearing joint surface. However, if the fracture is severely comminuted or crushed, good results are difficult to obtain with open reduction and internal fixation.

We report a femoral head fracture with a large, crushed defect successfully treated through a novel autologous osteochondral transplantation from the ipsilateral femoral head (repositionplasty).

## 2. Case presentation

**Figure 1.**
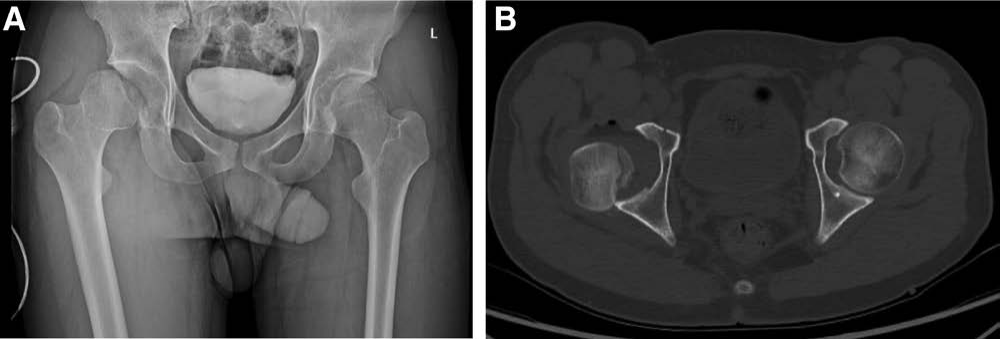
Initial image workup. (A): Anteroposterior hip x-ray showing right hip dislocation and femoral head fracture (pipkin type II). (B): Axial CT showing posterior dislocation of the right hip and a severely crushed femoral head. CT = computed tomography.

**Figure 2.**
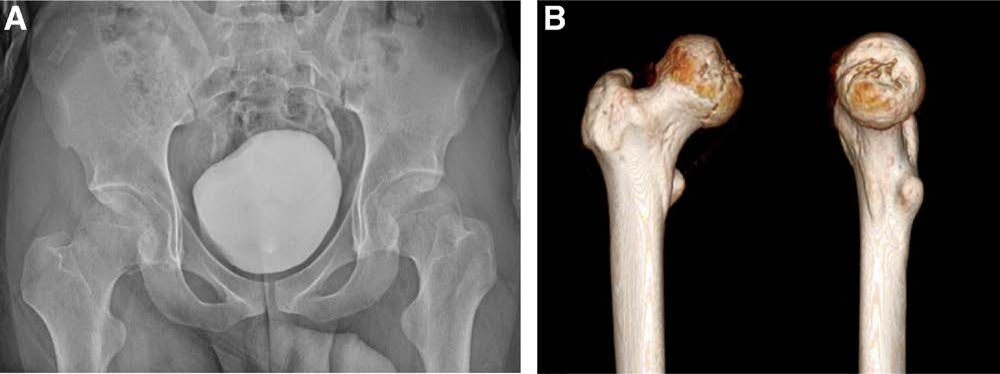
(A): Anteroposterior hip x-ray after reduction shows correct positioning of the femoral head. (B): 3-dimension reconstruction image of the femoral head showing a large defect in the weight-bearing surface.

**Figure 3.**
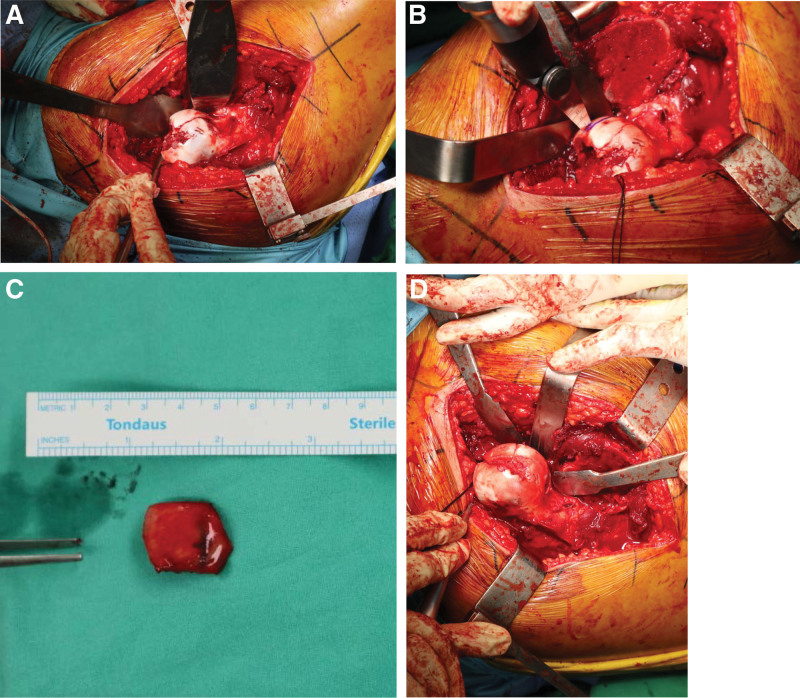
(A): Intraoperative image showing an approximately 2 × 3-cm defect in the superomedial side of the femoral head. (B): The osteochondral fragment was harvested from the inferomedial side of the femoral head using a microsaw. (C): The harvested osteochondral cartilage measured approximately 2 × 2.5 cm. (D): After transplantation, the articular surface of the femoral head appears congruent.

A 23-years-old male patient presented to the emergency room with right hip pain after 4-m fall. In the emergency department, he was diagnosed with right posterior hip dislocation and a Pipkin type 2 femoral head fracture with an ipsilateral superior ramus fracture (Fig. 1). After manual reduction, simple radiography and computed tomography showed reduction of the hip joint and osteochondral defects were observed in the anterior superior region of the femoral head due to impaction, such as a Hill–Sachs lesion in the shoulder (Fig. 2). Surgery was performed the day after the injury. The Kocher–Langenbeck approach was performed with the patient in the lateral position under general anesthesia. Flip osteotomy and surgical dislocation were performed to secure a wide field of view for accurate evaluation and reduction of intra-articular lesions.^[5]^ An osteochondral defect of approximately 2 × 3 cm was found in the anterior superior region of the femoral head, which is the weight-bearing part, and the fracture extended to the inferior medial side of the femoral head. Because the defect was large, the hip joint was unstable and could easily be dislocated posteriorly. Since the defect site could not be reduced with the original fracture fragment, osteochondral cartilage was harvested from the inferior medial side of the femoral head using a microsaw and transplanted into the defect site. The harvested fragments were confirmed to be congruent in several directions (Fig. 3). To create a congruent articular surface, the cancellous bone collected from the flip osteotomy site was inserted into the area where the graft appeared to be depressed, making the joint surface congruent.

**Figure 4.**
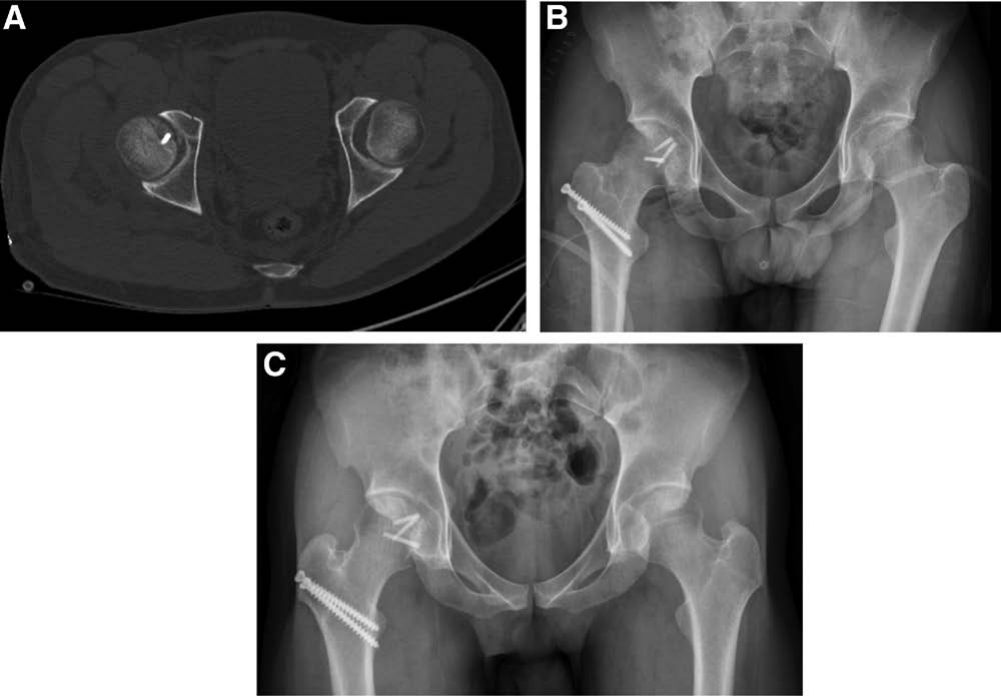
(A, B): Immediate post-operative computed tomography and Hip Anteroposterior x-ray showing a congruent femoral head. (C): Anteroposterior hip x-ray at 5 years postoperatively shows no evidence of collapse or arthritic change in the femoral head.

The graft was fixed using a headless screw (Acutrak Standard, Acumed, Hillsboro, OR) and the flip osteotomy with a 6.5 mm cancellous screw (Synthes, Oberdorf, Switzerland). After hip reduction, the instability disappeared. The ipsilateral superior ramus fracture was stable and conservative treatment was administered. Weight-bearing was not permitted for 4 weeks after surgery, and the patient was gradually allowed to use crutches. The congruent reduction was confirmed by radiography and computed tomography immediately after the surgery. At 5 years postoperatively, the Harris Hip Score was 100 points and no discomfort, femoral head collapse, osteonecrosis, or traumatic arthritis were observed on follow-up radiographs (Fig. 4). The patient provided informed consent to the data concerning his case being submitted for publication.

## 3. Discussion

Femoral head fracture is a rare type of fracture known to accompany hip dislocation, and occurs in approximately 6% to 16% of posterior hip dislocations.^[1–3]^ Various surgical methods are used to treat femoral head fractures, ranging from conservative treatment to fragment excision, open reduction and internal fixation, and prosthetic replacement. According to Giannoudis, fragment excision is mainly used in Pipkin type 1 fractures, where the size of the bone fragment is small.^[6]^ In the case of Pipkin type 2 fractures, because the bone fragments are large and the fracture is in the weight-bearing area, open reduction and internal fixation are most commonly performed. However, if the fracture is severely comminuted or crushed, it is difficult to fix, so a good prognosis would be unlikely. A small fragment in the non-weight-bearing area would not cause long-term clinical problems; however, a large defect in the weight-bearing area would change pressure distribution on the acetabulum and femoral head, leading to poor outcomes.^[7]^

Arthroplasty, osteochondral autologous transplantation (OAT), and mosaicplasty have been reported for treatment of osteochondral defects of the femoral head.^[8]^ Arthroplasty at a young and active age is not a good choice, because liner wear and osteolysis can easily occur, leading to a high reoperation rate.^[4]^ OAT is a method of harvesting osteochondral fragments using a cylindrical donor harvester and then transplanting them to an osteochondral lesion. It is mainly used for osteochondral lesions of the knee. Girard et al^[9]^ reported good results by transplanting osteochondral fragments harvested from the non-weight-bearing region of the femoral head in a young patient with an osteochondral lesion in the weight-bearing region of the femoral head. The author also reported good results using ipsilateral femoral head mosaicplasty for osteochondral defects after a femoral head fracture.^[10]^ Compared with OAT harvested from the knee, the procedure introduced in this case report has the advantages of a shorter operation time, less bleeding, and no donor site morbidity, because the bone fragments are harvested from the inferior part of the ipsilateral femoral head. Because the femoral head has a spherical structure, a graft from this area is theoretically closer to the proper anatomical shape than a graft from the knee. Restoring the anatomical shape is expected to reduce arthritic changes by more evenly distributing the pressure of the hip joint. In addition, large bone fragments can be freely harvested without being limited by the size of the cylinderlic donor harvester. Moreover, compared to mosaicplasty, there is no defect between the plugs, making it more advantageous in terms of pressure distribution, and the lower number of screws used, as not all plugs require screws. Since this method harvests osteochondral cartilage from the inferior part of the femoral head, it is necessary to study its effect on blood supply to the superior part of the femoral head. Owing to the short follow-up period of this report, additional studies on delayed complications are needed.

## 4. Conclusion

We have experienced excellent clinical and radiological results using a method we devised for treating large osteochondral defects associated with femoral head fractures. We believe this method will be a good treatment method for young patients with a large defect in the weight-bearing part of the femoral head who cannot undergo open reduction and internal fixation. However, as this is only a single case, with a short follow-up period, further studies are needed.

## Author contributions

**Conceptualization:** Hyun-Chul Shon.

**Data curation:** Jae-Young Yang.

**Formal analysis:** Jae-Young Yang.

**Investigation:** Hyun-Chul Shon, Jae-Young Yang.

**Supervision:** Eic-Ju Lim, Seung-Jun Jeon.

**Writing – original draft:** Jae-Young Yang.

**Writing – review & editing:** Eic-Ju Lim, Jae-Young Yang, Seung-Jun Jeon.
